# Phylogeny as a Proxy for Ecology in Seagrass Amphipods: Which Traits Are Most Conserved?

**DOI:** 10.1371/journal.pone.0057550

**Published:** 2013-03-07

**Authors:** Rebecca J. Best, John J. Stachowicz

**Affiliations:** Department of Evolution and Ecology, University of California Davis, Davis, California, United States of America; Swansea University, United Kingdom

## Abstract

Increasingly, studies of community assembly and ecosystem function combine trait data and phylogenetic relationships to gain novel insight into the ecological and evolutionary constraints on community dynamics. However, the key to interpreting these two types of information is an understanding of the extent to which traits are phylogenetically conserved. In this study, we develop the necessary framework for community phylogenetics approaches in a system of marine crustacean herbivores that play an important role in the ecosystem functioning of seagrass systems worldwide. For 16 species of amphipods and isopods, we (1) reconstructed phylogenetic relationships using COI, 16S, and 18S sequences and Bayesian analyses, (2) measured traits that are potentially important for assembling species between and within habitats, and (3) compared the degree to which each of these traits are evolutionarily conserved. Despite poor phylogenetic resolution for the order Amphipoda as a whole, we resolved almost all of the topology for the species in our system, and used a sampling of ultrametric trees from the posterior distribution to account for remaining uncertainty in topology and branch lengths. We found that traits varied widely in their degree of phylogenetic signal. Body mass, fecundity, and tube building showed very strong phylogenetic signal, and temperature tolerance and feeding traits showed much less. As such, the degree of signal was not predictable based on whether the trait is related to environmental filtering or to resource partitioning. Further, we found that even with strong phylogenetic signal in body size, (which may have large impacts on ecosystem function), the predictive relationship between phylogenetic diversity and ecosystem function is not straightforward. We show that patterns of phylogenetic diversity in communities of seagrass mesograzers could lead to a variety of interpretations and predictions, and that detailed study of trait similarities and differences will be necessary to interpret these patterns.

## Introduction

Ecologists are increasingly interested in using phylogenetic proxies for ecological variation among species, whether to understand community assembly [1], [2], [3] and ecosystem function [4], [5], or to guide conservation and restoration efforts [6]. For example, assuming that more closely related species are more similar ecologically, we might conclude that communities composed of close relatives were assembled according to their shared environmental tolerances or habitat requirements (environmental filtering). Conversely, communities composed of distantly related species might be structured by competitive exclusion, with only distant relatives being sufficiently divergent in their traits to partition resources [2]. As a corollary to the idea that resource use diverges among species over evolutionary time, higher phylogenetic diversity could also increase ecosystem function via complementarity or sampling of dominant phenotypes [4]. However, as many reviewers of this rapidly expanding field have pointed out, it is critical that we test, rather than assume, the extent to which important trait differences are correlated with phylogenetic distances [7], [8], [9].

Within a clade of species, traits vary in their degree of conservatism [7], [9], [10]. This is not surprising. Both environmental tolerance and resource partitioning traits must evolve in the same species. For this to happen, species are quite likely to undergo change in some traits while others stay the same, regardless of whether diversification along an environmental gradient or within a particular habitat type happens first [11], [12], [13], [14]. Differences in the level of functional constraint, the type of selection, and changing divergence rates over time can also all contribute to variation in the extent to which phylogeny predicts trait differences [15], [16], [17].

Although numerous studies have tested for phylogenetic signal in plant traits [4], we have much less information about the lability of important functional traits in animals, outside of a few very well studied groups (e.g., Caribbean lizards). In this study, we worked with a group of herbivorous marine invertebrates that represents an excellent potential system for community phylogenetic approaches but so far has lacked the necessary understanding of phylogenetic relationships and trait lability. In contrast to many marine communities, which involve interactions between animals from multiple phyla, the amphipods and isopods in our system represent approximately 83% of the mesograzer species in just 2 orders of peracarid crustaceans. Most of the coexisting species in a community are therefore closely related enough that meaningful phylogenetic and trait distances can be estimated. Species share fundamental morphological and life history features, such as mouthparts and direct development from eggs brooded by the female, although they vary quantitatively in these traits.

Additionally, amphipods and isopods represent an important guild of herbivores in seagrass meadows worldwide, consuming algae and detritus and increasing available light for photosynthesis by seagrasses, which in turn provide habitat for a diversity of economically important fish and crustaceans [18], [19], [20]. Because of these important habitat functions, and because coastal development threatens seagrass worldwide [21], these systems have been a key focus for studying the links between biodiversity and ecosystem function in marine systems [22]. To inform community phylogenetics approaches in this system we (a) reconstructed phylogenetic relationships between grazers, (b) measured a range of traits, and (c) determined the relative strength of phylogenetic signal in these traits. We discuss our findings in terms of their implications for studies using patterns of phylogenetic diversity to infer community assembly processes and predict ecosystem functioning.

## Methods

### Study System

We conducted our study in Bodega Bay, California (38° 19.110′N 123° 04.294′ W), collecting sequences and trait data for 14 species of amphipods and 2 species of isopods. These species are epifaunal, inhabiting seagrass beds and patches of macroalgae (*Ulva* spp.) growing on mudflats and floating docks (Table 1). They feed on epiphytic microalgae, macroalgae, eelgrass, and eelgrass detritus. Most species are found in the protected waters of Bodega Harbor, with 3 additional species collected from similar habitats (surfgrass beds and patches of *Ulva* spp. growing on the rocks) on the adjacent open coast (Table 1). This species pool allowed us to test for phylogenetic signal in species across a range of habitats that share some characteristics (such as algae or seagrass as habitat structure) and vary in others (such as water temperature). We collected individuals for all parts of this study under Scientific Collecting Permits issued by the California Department of Fish and Game.

**Table 1:** Species included in the study.

				Accession Numbers		
Family	Species	Authority	Habitat*	COI	16S	18S
*Suborder: Corophiidea*						
Ampithoidae	*Ampithoe dalli*	Shoemaker, 1938	OC	JX545453	JX545422	JX545386, JX545350
Ampithoidae	*Ampithoe lacertosa*	Bate, 1858	M, E	JX545454	JX545424	JX545388, JX545352
Ampithoidae	*Ampithoe sectimanus*	Conlan and Bousfield, 1982	M, E	JX545457	JX545427	JX545391, JX545355
Ampithoidae	*Ampithoe valida*	S. I. Smith, 1873	M, E	JX545459	JX545429	JX545393, JX545357
Aoridae	*Aoroides columbiae*	Walker, 1898	M, E, F	JX545451	JX545421	JX545385, JX545349
Aoridae	*Grandidierella japonica*	Stephense, 1938	M	JX545464	JX545436	JX545400, JX545364
Caprellidae	*Caprella californica*	Stimpson, 1857	E, F	JX545460	JX545430	JX545394, JX545358
Caprellidae	*Caprella mutica*	Schurin, 1935	F	**	JX545432	JX545396, JX545360
Ischyroceridae	*Ericthonius brasiliensis*	Dana, 1853	E	JX545462	JX545434	JX545398, JX545362
Ischyroceridae	*Ischyrocerus anguipes*	Krøyer, 1838	E	JX545466	JX545438	JX545402, JX545366
*Suborder: Gammaridea*						
Dogielinotidae	*Allorchestes angusta*	Dana, 1856	M	JX545449	JX545418	JX545382, JX545346
Hyalidae	*Parallorchestes cowani*	Bousfield & Hendrycks, 2002	OC	JX545470	JX545443	JX545412, JX545376
Hyalidae	*Protohyale frequens*	Stout, 1913	OC	JX545472	JX545445	JX545414, JX545378
Pontogeneiidae	*Pontogeneia rostrata*	Gurjanova, 1938	E	JX545474	JX545447	JX545416, JX545380
*Order: Isopoda*						
Idoteidae	*Idotea resecata*	Stimpson, 1857	E	JX545469	JX545441	JX545405, JX545369
Sphaeromatidae	*Paracerceis cordata*	(Richardson, 1899)	M, E, F	**	JX545442	JX545410, JX545374

* OC = outer coast, M = drift algae on mudflats, E = eelgrass bed, F = fouling community (M, E, F = harbor habitats).

** COI sequences for these 2 species could not be obtained after 5 attempts. For *Caprella mutica*, we substituted a randomly selected COI sequence available on GenBank (GU130250). For *Paracerceis cordata*, this gene was not represented in the analysis.

### Phylogeny

Because the 16 species in this study are sparsely sampled from 9 different families (7 amphipod families and 2 isopod families), we used two sources of data to construct the phylogeny. First, we sequenced our 16 local species, using 2 individuals of each species for portions of 3 genes: mitochondrial cytochrome *c* oxidase subunit I (COI) and 16S rRNA, and nuclear 18S rRNA. The 18S gene has been widely used in crustacean phylogenetics generally, and in peracarid [23], isopod [24], and amphipod phylogenetics in particular [25], [26], [27]. This combination of 3 genes has also been used previously in a study of Lake Baikal amphipods [28].

Second, we searched for additional sequences on GenBank ([29] accessed May 2011) from amphipods in the two major suborders represented in our system: Corophiidea and Gammaridea. We used the following search criteria: we included only one sequence per species, excluded taxa only identified to genus (except when they were the only available representatives of their family), and excluded endemic freshwater families. We included up to 3 species per family if available, from as many different genera as possible, except for a few families particularly important in our study system, for which we included up to 6 species. This search yielded 74 additional 18S sequences (4 for species in our system plus 70 additional species) and allowed us to include at least one representative of 41 amphipod families (32 of 45 families present in our region [30]). Unfortunately, few of these additional species had available mitochondrial sequences. For the larger pool of species we therefore focused on 18S only; species and accession numbers for this data set are given in Table S1. We present the single gene analysis only for clarity of interpretation, but obtained similar results from a data matrix using all 3 genes with a high proportion of missing data.

#### Molecular methods

To sequence the species in our system, we collected individuals in the summers of 2009 and 2010, preserved them in 100% EtOH, and isolated DNA using DNeasy Blood and Tissue kits (Qiagen Inc., Valencia, CA). We then amplified each gene using PCR; primers are given in Table 2. All PCR reactions were 25 µL and run on a GeneAmp PCR System 9700 (Applied Biosystems [AB], Foster City, CA). For 16S and COI, reactions consisted of 0.25 µL of Amplitaq Gold DNA Polymerase, 2.5 µL of 10× Amplitaq Gold Buffer, 2 µL of 25 mM MgCl, and 2.5 µL of 2 mM dNTPs (all AB), plus 0.5 µL of each 10 µM primer and 5 µL of ∼10 ng/uL DNA template. For 18S, the reactions instead had 0.5 µL of polymerase and 1.5 µL of 25 mM MgCl (all other components the same). For 16S and COI the PCR program was 4 min at 95°C; followed by 45 cycles of 1 min at 95°C, 1 min at 45°C, and 2.5 min at 72°C; followed by 7 min at 72°C. For 18S the PCR program was 5°min at 95°C; followed by 35 cycles of 20 s at 95°C, 20 s at 50°C, and 45 s at 72°C; followed by 7 min at 72°C. Recipes and PCR programs were adapted from [26] and [28]. PCR products were cleaned by combining 20 uL of product with 10 uL of sterile water, 0.5 uL of Exonuclease I and 1 uL of Shrimp Alkaline Phosphatase (both USB, Cleveland, OH) and running the reaction for 15 min at 37°C and 15 min at 80°C. Cleaned products were sent to the UC Davis UCDNA Sequencing Facility, which uses ABI 3730 Capillary Electrophoresis Genetic Analyzers and BigDye® Terminator v3.1 Cycle Sequencing Kits (both AB). Both forward and reverse sequences were obtained for each gene, and were used to confirm uncertain bases where present. All sequences were submitted to GenBank (see Table 1 for accession numbers).

**Table 2:** Genes sequenced and aligned for phylogenetic analysis.

Gene	Primer*	Sequence (5′-3′)	Length	Full align.	Clipped align.
18S	18SF [^26^]	CCTAYCTGGTTGATCCTGCCAGT	476–781	1209	465
	18S700R [^26^]	CGCGGCTGCTGGCACCAGAC			
	18S1250F [^26^]	CCGTTCTTAGTTGGTGGAGCG	568–894	1309	433
	18SR [^26^]	TAATGATCCTTCCGCAGGTT			
16S	16STf [^28^]	GGTAWHYTRACYGTGCTAAG	429–460	625	311
	16Sbr-H [^91^]	CCGGTTTGAACTCAGATCATGT			
COI	LCO1490 [^92^]	GGTCAACAAATCATAAAGATATTGG	710	710	710
	HCO2198 [^92^]	TAAACTTCAGGGTGACCAAAAAATCA			

* Primers are the same as those used in [28], with the exception of 1250F, which we substituted for 1500F.

#### Phylogenetic analysis

We aligned the sequences separately for each of 3 genes. For COI, we used the Muscle Alignment [31] in Geneious [32] with default settings and inspected the translated protein sequences for frame shift errors. No insertions or deletions were necessary (Table 2). For the 16S and 18S rRNA genes we used SSU-ALIGN 0.1 [33] to obtain secondary-structure guided alignments. For 18S, we used the eukaryote secondary structure model for small subunit rRNA which is provided with the SSU-ALIGN program. For 16S, we first built the secondary structure model for our portion of the 16S gene from the secondary structures for 11 invertebrate mitochondrial large subunit rRNA sequences available on the Comparative RNA Website (also the source for SSU-ALIGN’s default secondary structure models [34]). We used two iterations of model building as described in the SSU-ALIGN manual, and found the structure to be highly similar between the seed sequences, which included 1 annelid, 2 arthropods, and 8 mollusks. For both 16S and 18S, we used the mask function in SSU-ALIGN to retain only those sites where at least 85% of the sequences had probabilities above 85%. In preliminary tests this limit appeared to substantially increase the number of included sites (in comparison to 95% limits), without greatly decreasing accuracy [35]. Sequence lengths, and the lengths of the full and retained alignments, are given in Table 2.

We first constructed the phylogeny using only the sequences for our 16 species with trait data. We used Bayesian modeling in MrBayes [36] with five partitions: one for the COI gene, and one for each of the stem and loop portions of 16S and 18S. We used the GTR+Gamma model for the COI and loop partitions because preliminary analyses in MrBayes indicated all GTR parameters could be estimated. We also found that the GTR+Gamma model returned the highest support scores using MrModeltest [37]. We used the doublet model [38] for the stem sections of rRNA, taking the doublet pairs from the alignment output by SSU-ALIGN. We allowed substitution rates, the gamma shape parameter, state frequencies, and the GTR model parameters to vary across partitions, and retained default priors for these parameters with the exception of the state frequencies for the rRNA stem partitions, which were set as empirical. Using these parameters, we ran the MCMCMC analysis with 4 chains, a temperature of 0.02, and 2 swaps per generation for 10 million generations, sampling every 1000. In initial analyses we included both sequences for each species, but average pairwise similarity within species was 98% for COI and 16S and 99.9% for 18S, and replicate sequences always grouped together with 100% certainty. We therefore randomly selected only one individual per species to use in all further analyses.

After completing two simultaneous runs in MrBayes, we used Tracer [39] to confirm adequate effective sample size, mixing, and stationarity of each parameter in each run, as well as convergence between runs, after a 10% burn-in period. We also ensured that acceptance probabilities for parameters and chain swaps were between 10 and 70%, which was not the case using the default temperature of 0.2. We repeated this analysis for the mitochondrial (16S and COI) and nuclear (18S) genes separately, with the only change being that we increased the temperature to 0.1 in both cases. Finally, we used the same model and MCMCMC settings to reconstruct a full phylogeny for all 88 species, including the 18 from our system (the 16 for which we have trait data plus two additional rare species we sequenced, see Table S1) and 70 from GenBank, based on only the nuclear 18S gene. For this larger dataset we ran analyses using the CIPRES supercomputing resource [40]. For all MrBayes analyses, we report results from a single execution of the 2-run analysis. However, in the process of optimizing MCMC settings we observed that within a particular model, all analyses reaching convergence (2–4 independent analyses) did sample from the same final distribution. We also ran each model with no data to sample the prior distributions for each parameter to confirm that the priors were not driving the results.

To test hypotheses about trait evolution through time, we required ultrametric trees. For just the 16 species for which we had trait data, we obtained a posterior distribution of ultrametric trees with relative branch lengths using BEAST [41]. We again used 5 partitions (the COI gene and the stem and loop portions of each of the 16S and 18S genes). Because the doublet model for rRNA stems is not implemented in BEAST, we used unlinked GTR+Gamma models for each partition. To ensure adequate mixing of all parameters, we found it necessary to use lognormal rather than gamma priors for the 6 GTR rates describing both 16S partitions and the 18S stem partition. This is because the default gamma priors in BEAST do not accommodate near-zero values of any of the rate parameters. We chose to use the alternate priors rather than switch to a reduced HKY model because the parameters fit during the MrBayes analysis indicated more variation in substitution rates than just a difference between transitions and transversions. We used a Yule speciation process tree prior, a random starting tree, the constraint that both the isopod outgroup and the amphipod ingroup be monophyletic (based on support from the MrBayes analyses), and a lognormal uncorrelated relaxed clock. The earliest fossil isopod dates from the Pennsylvanian (∼ 300 million years ago) [42], and the amphipods are thought to have diverged some time after that, although the earliest accepted specimens date from only 30–50 million years ago [43]. Because time calibration data is not available, we left the clock mean hyperparameter fixed at 1.0 to obtain relative branch lengths.

We ran 3 independent analyses with these settings, for 20, 15, and 15 million generations, sampling every 1000 generations. We assessed the performance of these runs and compared them to each other and to the prior using Tracer, as described for the MrBayes analyses. We also confirmed that the posterior distribution for the standard deviation of the clock rate across the tree did not include 0, supporting the selection of a relaxed rather than strict clock. After ensuring that the multiple analyses had converged on the same stationary distribution after a 10% burn-in period, we removed the first 2000 trees from each run and combined the rest to give a single posterior distribution of 44,000 trees. From this distribution we determined the maximum clade credibility tree with posterior probabilities for each node. We then randomly sampled 1000 trees from this distribution and used these for all trait evolution analyses.

### Traits

For each of the 16 species in our study, we measured a range of traits connected to morphology, life history, resource use, and environmental filtering. We measured size, fecundity, tube-building ability, and stable isotope signatures using field collections, and used laboratory assays at the Bodega Marine Lab to measure temperature tolerance and feeding rates. All trait measurements were made on adults of each species (defined on the basis of size and secondary sexual characters: egg production in females and gnathopods in males, as detailed in [30]).

#### Size

Because gammarids, caprellids, and isopods vary substantially in their shape, we used dry biomass as our index of size. For each species, we collected adults of both sexes in July 2011 and dried them at 60°C for 48 hours before weighing them. We used 10 replicates per species (5 of each sex), with 1 individual per replicate for the 10 larger species and 5 individuals per replicate for the 6 smallest species (dividing the dry biomass by 5).

#### Fecundity

We measured fecundity as the number of eggs per brooding female. We counted egg number for 7–15 brooding females of each species, minimizing the opportunity for egg mortality as much as possible by rejecting all females where any eggs were showing embryo development. We collected the brooding females as part of a year-long survey effort, ensuring that each species was represented by individuals collected in spring, summer, fall, and winter. This is important because brood size can vary within amphipod species over seasons [44], [45].

#### Tube building

Tube building is a major dimension of habitat use for amphipod species, with some species regularly found in tubes they have built in macroalgae, on eelgrass blades, or on the sediment surface. Amphipods build tubes using silk-producing glands on the 3^rd^ and 4^th^ pereopods, or walking legs [46]; species without these glands are never found in tubes and are not capable of building them. We observed which species are found in tubes when collecting them from the field, and confirmed this by watching tube construction in the lab.

#### Temperature tolerance

The species in this study are distributed across eelgrass habitats that range in depth from 3 to 0 m below mean lower low water (MLLW), and mudflat habitats from 0 to 0.7 m above MLLW. Because of this depth gradient, there is also substantial variation in water temperature. From a winter minimum around 5°C, summer water temperatures rise to a maximum of 17–19°C in deep eelgrass beds, fouling communities, and on the outer coast, and a maximum of 25–30°C in the shallow water covering mudflats during low tides in summer and fall (based on continuous measurement of water temperature using HOBO Pendant data loggers [Onset Computer Corp, Pocasset, MA] at all harbor habitats, and on the Bodega Ocean Observing Node [BOON] data for the outer coast [Data provided by the University of California, Davis, Bodega Marine Laboratory]).

To measure the kind of temperature tolerance that might affect species distributions in the field, we assessed mortality rates for each species under constant high temperature of 25°C relative to an average summer water temperature control of 12–15°C. We conducted these assays with animals collected in the summer because this is when animals might reasonably experience these temperatures. We used two 0.9 m x 0.75 m water tables with aquarium heaters maintaining a temperature of 25±0.1°C for the treatment, and a single 2.45 m x 0.75 m water bath with a constant flow of ambient temperature seawater for the control. Both water tables were in the same indoor wet lab on a 12 hour light/dark cycle. We also used a variable stress treatment, in which temperatures switched from ambient to elevated (25°C) every 12 hours to measure the kind of temperature acclimation stress that might be experienced with tidal fluctuations. However, the results of this treatment were highly correlated with the constant stress treatment (r = 0.84, p<0.0001), and showed the same amount of phylogenetic signal so we do not present them here.

We put single individuals of a single species into individual 470 mL cups filled with seawater and placed these in the water tables. We used 15 replicates per species per treatment, and ensured that for each species we included a range of adult sizes, an equal sex ratio in control and treatment, and no brooding females. Individuals were collected from the field and held with food at ambient temperatures for approximately 5 days before each experiment. In order to accommodate the total number of replicates needed (15×3 treatments x 16 species = 720), we used a total of 6 sequential trails between August 1 and 27, 2011, running each trial for 4 days. We recorded the time to death for each replicate at 12 hour intervals, up to a maximum of 96 hours, and calculated the mean effect of the treatment vs. control on time to death for each species. Our measure of temperature tolerance is thus the average reduction in survival time under elevated temperature. To test the significance of the treatment effect in each species we used survival analysis, specifically a log-rank test for differences in the timing of events (deaths), implemented using the survdiff function in the Survival package [47] in R [48]. All species with p-values for treatment effects greater than 0.05 were given values of 0 for this trait to indicate that elevated temperature had no effect on survival time. We also examined this criterion using a multiple comparison adjustment of p = 0.05/16 species; this had no effect on significance determinations.

Because it was not possible to simultaneously collect and test all 16 species, each trial tested a new set of 3 species representing a variety of habitats. Although water temperatures in the 25°C water baths were tightly regulated and temperatures in the control did not vary substantially among trials, there is still some possibility of confounding differences among species with differences among trials. To assess this, we ran a 7^th^ trial immediately following the others in which we re-tested 6 species that are found in a range of habitats, were tested in different trials, and varied in their response to elevated temperature. We again used 15 replicates for each species, and found a strong correlation between the effects of increased temperature measured in separate trials compared to the group trial (r = 0.96, p = 0.002), so we do not consider variation among trials to be significant.

### Feeding Traits

We measured feeding rates for each species using 48-hour no-choice feeding trials. We placed 2–4 individuals of a single grazer species in a 250 mL plastic cup filled with seawater, starved them for 24 h to standardize hunger levels, then added a single food: eelgrass, eelgrass detritus (tissue collected live and aged 4 weeks in flow-through seawater in the dark), macroalgae (*Ulva* spp.), or epiphytic microalgae. The species-specific number of individuals per cup was necessary to ensure measurable consumption within the fixed duration of the experiments. We used a total of 5 separate trials, 2 in the summer of 2009, two in the summer of 2010, and one in the summer of 2011, with the replicates for each species combined with each food split between at least two trials. In total we obtained 6–10 replicate cups per food per grazer species, after rejecting replicates with grazer mortality. To account for growth or decay of food items we used 10 no-grazer controls for each food in each trial. All trials were conducted in an indoor wet lab on a 12 hour light/dark cycle.

We measured consumption of the macrophytes (eelgrass, detritus, and macroalgae) as change in wet weight, starting with an approximately 2 cm^2^ piece of food and adjusting the starting weight by the average percent change in control cups for that food in that trial (see [47] for more details). We quantified microalgae consumption by offering grazers 9 cm^2^ pieces of window screen covered with microalgae (grown in the field) and measured consumption as the reduction in chlorophyll a (hereafter chla) relative to no-grazer controls for that trial (for detailed chlorophyll measurement methods see [49]).

To estimate feeding rates we used a mixed effects model for each food type (macroalgae, eelgrass, detritus, and microalgae), with amount eaten per individual per 24 hours as the response variable, grazer species as the fixed effect, and trial as the random effect. We conducted this analysis in SAS [50] using the MIXED Procedure with the Kenward-Roger method for estimating denominator degrees of freedom [51]. All residuals were checked for adherence to assumptions of normality and equal variance and no transformations were necessary. We considered individual feeding rates to be significantly different from 0 only if the p-value for that estimate was less than 0.05; otherwise feeding rates were recorded as 0.

### Stable Isotope Signatures

As an additional, field-based indicator of food use that should reflect actual rather than potential diet, we obtained carbon (δ^13^C ) and nitrogen (δ^15^N ) stable isotope ratios for each grazer species. Carbon and nitrogen isotopes have previously been used to detect feeding differences among amphipod species [52], [53], [54], and, with varying success, to distinguish between macroalgae, benthic microalgae, phytoplankton and vascular plants [55], [56], [57], [58]. We measured these for each of the harbor species in both winter (December 2009) and summer (July 2010), with one exception: *Caprella mutica* is largely absent in the winter and therefore could not be collected in that season. We do not analyze stable isotope ratios for outer coast species because the stable isotope signatures of outer coast primary producers differed from those of the harbor primary producers (even for the same type of macroalgae, *Ulva* spp.). The isotopic ratios of the species from these two different locations were thus not comparable as a measure of overlap in the realized diet. We collected 3 replicates per species per season, spreading collections over the multiple field sites where each species is found. Replicates required only 1 individual for large species, and several individuals for small species. We held all animals live in seawater with no food for 24 hours to ensure gut evacuation, rinsed them in deionized water, and placed them in a drying oven at 40°C for 24 hours. To compare the grazer signatures with the primary producers available in Bodega Harbor we also collected 3 to 5 samples each of fresh eelgrass tissue, eelgrass detritus, *Ulva* macroalgae, and epiphytic microalgae. Similar to the feeding trials, we harvested the epiphytes from window screen anchored in an eelgrass bed for 2 weeks to avoid contamination with eelgrass detritus. We cleaned all primary producer samples under a microscope, rinsed them in deionized water, and dried them as above. For the epiphytes only, complete separation of the dominant microalgae (diatoms) from colonizing animals (mostly nematodes) was difficult, and some component of animal tissue is likely. We homogenized each sample with a mortar and pestle cleaned with methanol, and submitted samples for analysis at the UC Davis Stable Isotope Facility. The δ^13^C and δ^15^N signatures obtained give the isotope composition relative to the international standards of V-PDB and air, respectively.

### Phylogenetic Signal

Tests for phylogenetic signal in trait data depend first on whether the trait evolves continuously along an axis or exists only as discrete states with intervening transition rates. In our study, size, fecundity, δ^13^C and δ^15^N signatures, and temperature tolerance are clearly continuous traits, and tube-building ability is clearly a discrete trait. However, evolution in feeding behavior could follow either process. Discrete changes in mouthpart morphology, digestive physiology, or behavior might be necessary for a species to use a new food resource. However, increased use of particular resources could result from more quantitative continuous change in morphology, physiology or behavior. To accommodate both possibilities, we tested for phylogenetic signal in the continuous feeding rates for each food (macroalgae, eelgrass, detritus, and epiphytic microalgae), and for the discrete consumption vs. no consumption of each food.

For continuous traits we used two methods: Blomberg’s K [15] and Pagel’s λ [59], both implemented in the Phytools package [60] in R [48]. For discrete traits (tube building, food use), we used Pagel’s λ, implemented in the Geiger package in R [61] and assuming equal transition rates in both directions. Pagel’s λ varies from 0, where a star phylogeny or polytomy best represents the trait relationships among species, to approximately 1, where the actual phylogeny best represents the trait relationships. Blomberg’s K varies from 0 (no correspondence between phylogeny and the trait) to 1 (evolution by Brownian motion, wherein trait differences are correlated with the time available for them to develop via random divergence), to greater than 1 (closely related species have diverged in phenotype even less than expected based on the amount of time they have been separated).

Our objective with this study was to determine whether some traits in our species are better represented by phylogenetic proxies than others. For this reason, and because phylogenies with fewer than 20 taxa have reduced power to detect significant signal [15], we focus on the relative evidence of signal among traits more than binary conclusions that particular traits do or do not have signal. In all cases, we compared both the estimates for the parameter values (K and λ) and the results of significance tests, with smaller p-values indicating a more reliable correlation between that trait and the phylogeny. For K, the significance test compared the observed K to that obtained in 1000 randomizations of the trait values on the tree, and for λ, the significance test was a likelihood ratio test comparing the likelihood with the fitted value of λ to that with λ = 0. Using the Blomberg’s K approach in Phytools, which implements the method of [62], we also incorporated standard errors associated with the continuous trait means in our estimation of phylogenetic signal. However, we found that this had very little effect on estimates of K or p, and so report results using means only.

We repeated all analyses over 1000 trees sampled from the posterior distribution of ultrametric trees, and present the resulting distributions of λ, K, and the associated p-values. This has the major advantage of incorporating, rather than ignoring, uncertainty in topology and branch lengths, and provides a clear picture of the effect this uncertainty has on estimates of phylogenetic signal. This is in contrast to the use of polytomies to represent topological uncertainty, which can lead to overestimation of phylogenetic conservatism [63]. Finally, to test the sensitivity of our results to choices about the species pool, we repeated all analyses with two subsets of species: (1) all species found in harbor habitats only (i.e., excluding the three outer coast species), and (2) all amphipod species only (i.e., excluding the two isopod species).

## Results and Discussion

### Phylogenetic Reconstruction

**Figure 1:**
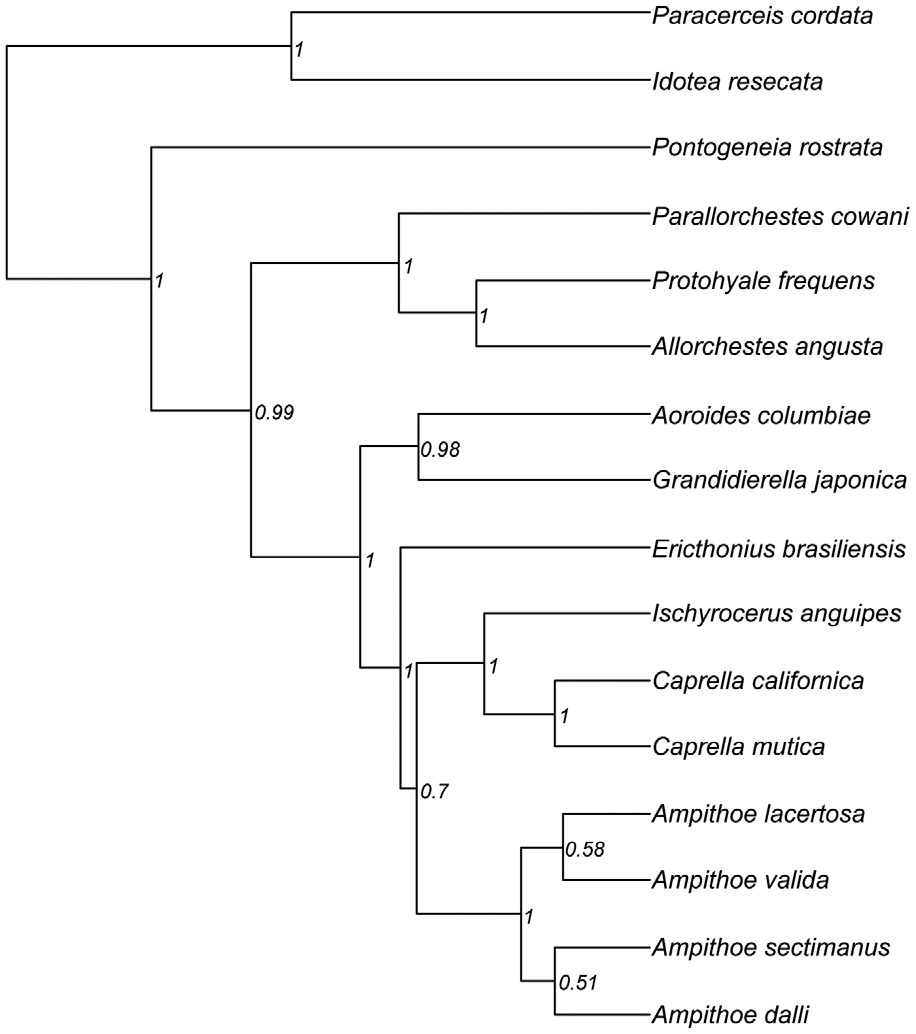
Maximum clade credibility ultrametric phylogeny for the 16 Bodega Bay species. Obtained from BEAST analyses using all genes (COI, 16S, 18S), branch lengths are in uncalibrated (relative) time units. Node labels are posterior probabilities. The isopods (*Paracerceis cordata* and *Idotea resecata*) are the outgroup.

The posterior distribution of ultrametric trees we obtained from the BEAST analysis is summarized by the maximum clade credibility phylogeny in Figure 1. For our subset of 16 species, deep splits in the topology were very well supported, with posterior probabilities of 1 or nearly so. The two major areas of uncertainty are (a) the relationships within the genus *Ampithoe*, and (b) the position of *Ericthonius brasiliensis*. *E*. *brasiliensis* is classified in the Ischyroceridae family with *Ischyrocerus anguipes* but was grouped frequently with the Ampithoidae, with the *Caprella*+*Ischyrocerus* group, or with both, as shown (Figure 1). Our ability to perform trait evolution analyses over a distribution of topologies representing each of these possibilities is a key strength of the Bayesian approach to phylogenetic reconstruction. Parsimony or maximum likelihood methods would indicate a similar lack of support for the marginally most likely option, without providing a means for integrating over the alternatives.

**Figure 2:**
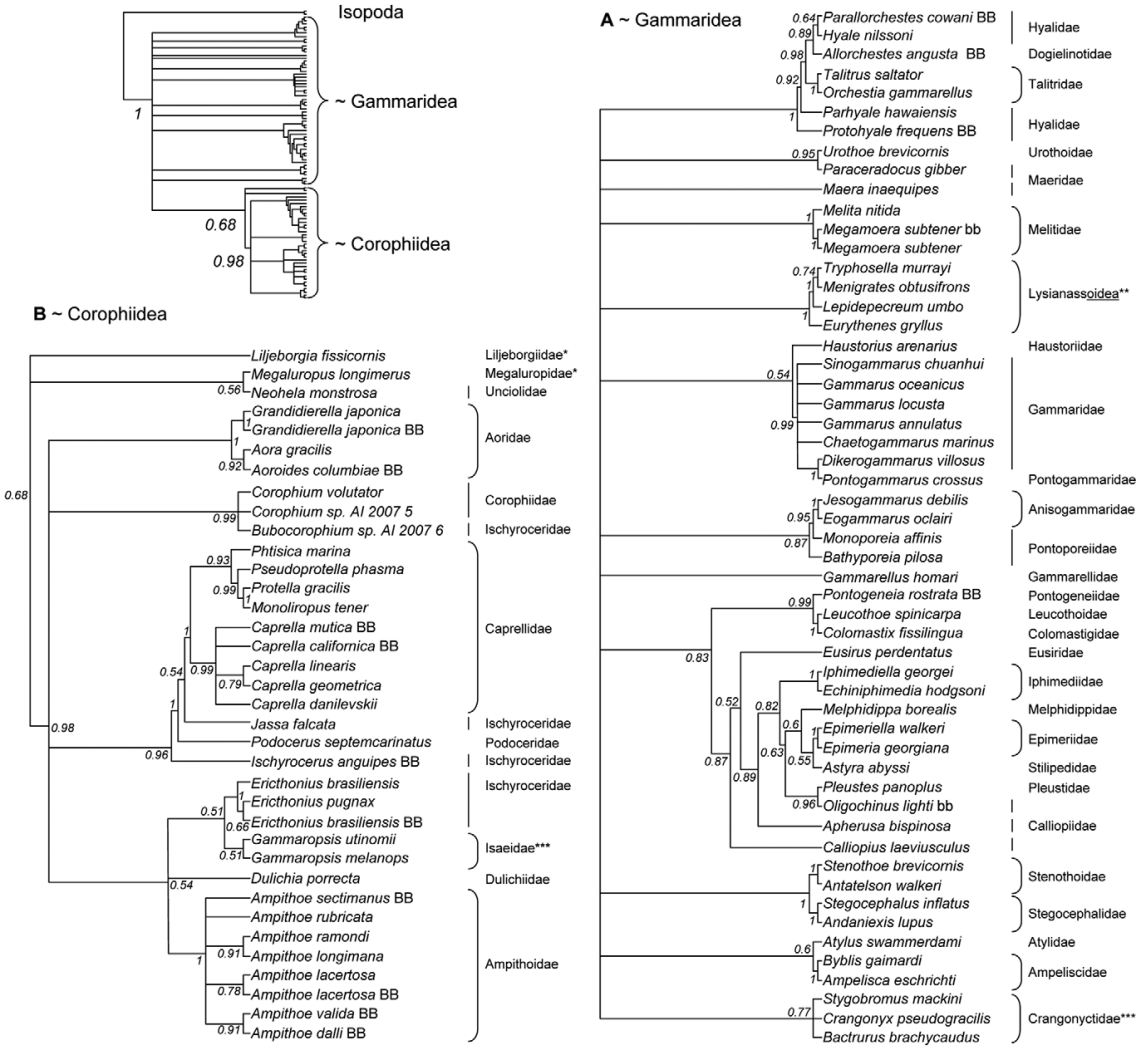
50% majority rule consensus cladogram for all 88 species based on the nuclear gene 18S. Node labels give posterior probabilities. Branch lengths are not meaningful. As shown in the upper left diagram, the cladogram consists of the Isopoda outgroup, and two subsections: A) suborder Gammaridea, and B) suborder Corophiidea. Two species (*) are grouped with the Corophiidea (although with low support), but are classified as Gammaridean. Rounded brackets show families with monophyletic topologies, plus the monophyletic superfamily Lysianassoidea (**). Monophyly brackets are supported with probability >0.99 with the exception of two families marked with ***. Non-monophyletic families are marked with vertical lines; families with no marking are represented by 1 species. New sequences from Bodega Bay are marked with BB for the 14 amphipod species with trait data and bb for the 2 without.

The overall high resolution obtained for our subset of species did not extend to the full sample of 88 species (Figure 2). Across this larger sample of species we found that the nuclear 18S rRNA gene did recover monophyletic relationships for some family groupings, and did recover the split between Corophiidean suborder (monophyletic) and Gammaridean suborder (paraphyletic), but provided little intermediate resolution between those two taxonomic levels (Figure 2). For the few intermediate taxonomic levels currently accepted [64] there is mixed support. Within the Corophiideans, neither the superfamily Caprelloidea (Caprellidae+Podoceridae+Isaeidae+Dulichiidae) nor the infraorders Corophiida and Caprellida [46] are supported, but the genus *Caprella* is monophyletic relative to other Caprellidae species. Of the Gammaridean superfamilies Lysianassoidea, Talitroidea (Talitridae+Hyalidae+Dogielinotidae), and Eusiroidea (Calliopiidae+Eusiridae+Gammarellidae+Pontogeneiidae) [65], only the first two are monophyletic.

This uncertainty is in keeping with much previous work on amphipod phylogenetics, which has mostly relied on morphological characters and has produced a series of different proposals for taxonomic groupings below the level of suborder [66], [67], [68]. A major attempt to resolve this uncertainty using 18S [69], which is the source of many of the GenBank sequences we included, also reached conclusions similar to ours despite substantial differences in methodology. Whereas the earlier work used maximum parsimony and quartet puzzling only [69], we used Bayesian methods to incorporate and evaluate uncertainty in parameter-rich models. The rapid evolution of computational resources and software also allowed us to incorporate secondary structure in the 18S gene both during alignment (increasing the probability of correctly identifying homologous positions) and during phylogenetic reconstruction. This is important because different rates of evolution in loop and stem regions and the non-independence of linked nucleotides in stem regions can both affect phylogenetic inference [70], [71], [72], [73]. However, these advances served only to increase our confidence in well-supported clades; they did not resolve the deep polytomies in the Amphipoda.

**Figure 3:**
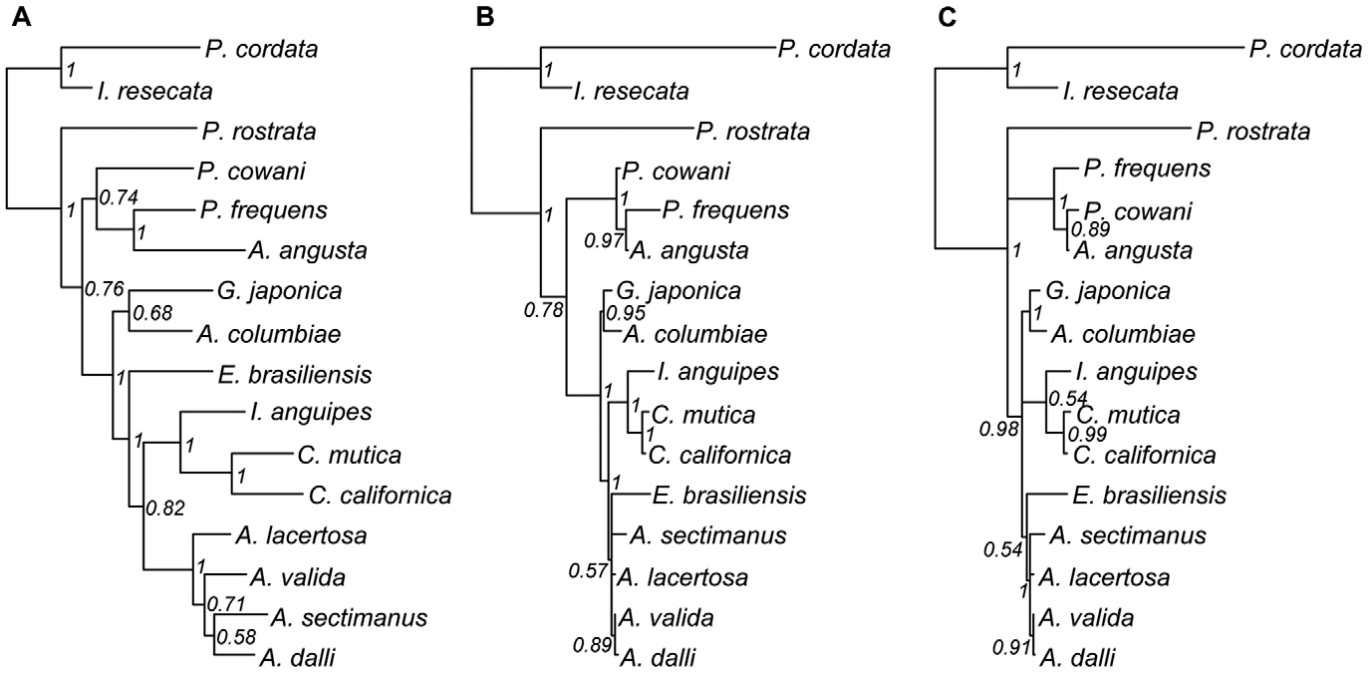
Comparison of phylogenies obtained for the 16 Bodega Bay species. A) mitochondrial gene tree (COI and 16S), B) nuclear gene tree (18S), C) topology for Bodega Bay species extracted from the 88 species 18S tree shown in Figure 2. All trees are 50% majority rule consensus trees from MrBayes analyses, with the node labels giving posterior probabilities. For full species names see Figure 1 (note that multiple genera with the same initial letter are abbreviated here). The only conflict between these topologies is within the Talitroidea (*Protohyale frequens*, *Parallorchestes cowani*, and *Allorchestes angusta*). However, the alternate topology obtained in the 88 species analysis is not well supported; the posterior probability = 0.89, where strong support is typically >95% for posterior probabilities (rather than >70% for bootstrap probabilities [89], [90]).

Beyond the 18S gene, our addition of COI and 16S for the species in our study did not have large effects on topology (Figure 3). Using the separate MrBayes analyses for COI and 16S vs. 18S, we reconstructed phylogenies with very similar topologies and no supported conflicts (Figure 3a,b). When using only 18S, we also obtained similar topologies for our 16 species with or without the inclusion of the 72 additional species (Figure 3b,c). However, the combination of all three genes did increase support for some important nodes, such as the *Ampithoe* genus and the early split of *Pontogeneia* from the other amphipods. The mitochondrial genes contained more information about change within families and genera than 18S, which produced very short relative branch lengths at this level (Figure 3). However, much of the resolution in the topology for our Bodega Bay species is likely due to the taxonomic sampling of the species found in our system. Alone, 18S recovered the major nodes within our topology (Figure 3b,c), but would not be able to produce similar resolution for a sampling of species from only the Gammaridean suborder, for example (Figure 2). Resolving those relationships, to the extent that it is possible, is likely to require a much more extensive addition of new markers.

### Phylogenetic Signal

**Figure 4:**
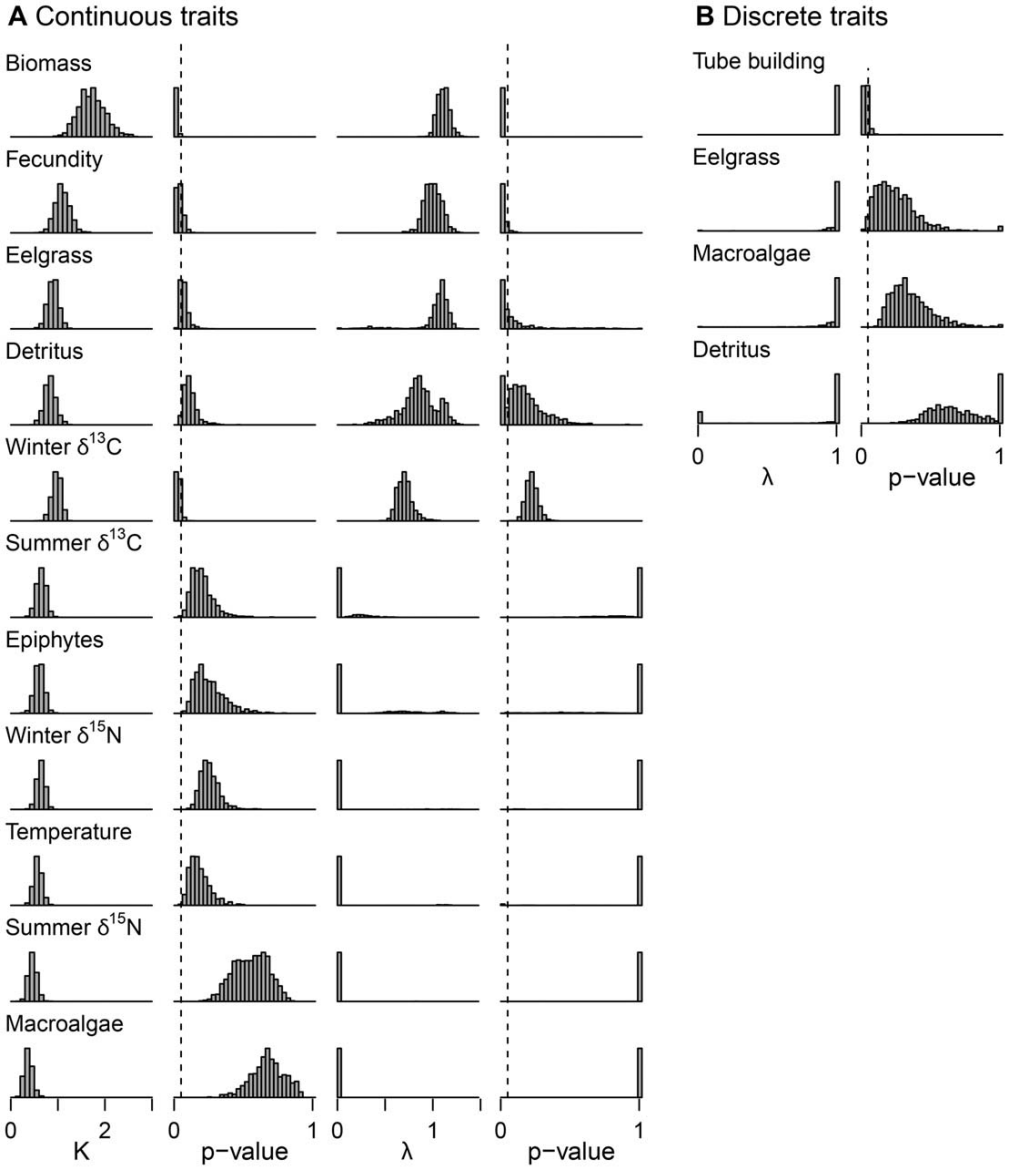
Relative phylogenetic signal in A) continuous, and B) discrete traits. For continuous traits, signal was assessed using both Blomberg’s K and Pagel’s λ, with significance tests for each. For discrete traits, signal was assessed with Pagel’s λ. The dashed lines indicate the p = 0.05 significance threshold for each test. The distributions of K, λ, and their p-values result from testing for phylogenetic signal across 1000 trees sampled from the Bayesian posterior distribution of ultrametric trees. Within the continuous and discrete categories, traits are ordered top to bottom from most to least evidence for phylogenetic signal. Signal in continuous feeding rates for eelgrass, detritus, and epiphytes decreased when examined on a per-mg of grazer basis (eelgrass: mean K decreased [0.9 to 0.7], mean λ decreased [1.0 to 0.8]; detritus: K decreased [0.8 to 0.5], λ decreased [0.9 to 0.02]; epiphytes: K decreased [0.6 to 0.5], λ decreased [0.3 to 0.03]; p-values for all tests increased). Results were opposite for macroalgae (mean K increased [0.4 to 0.6], mean λ increased [0 to 0.3], and p-values decreased).

**Figure 5:**
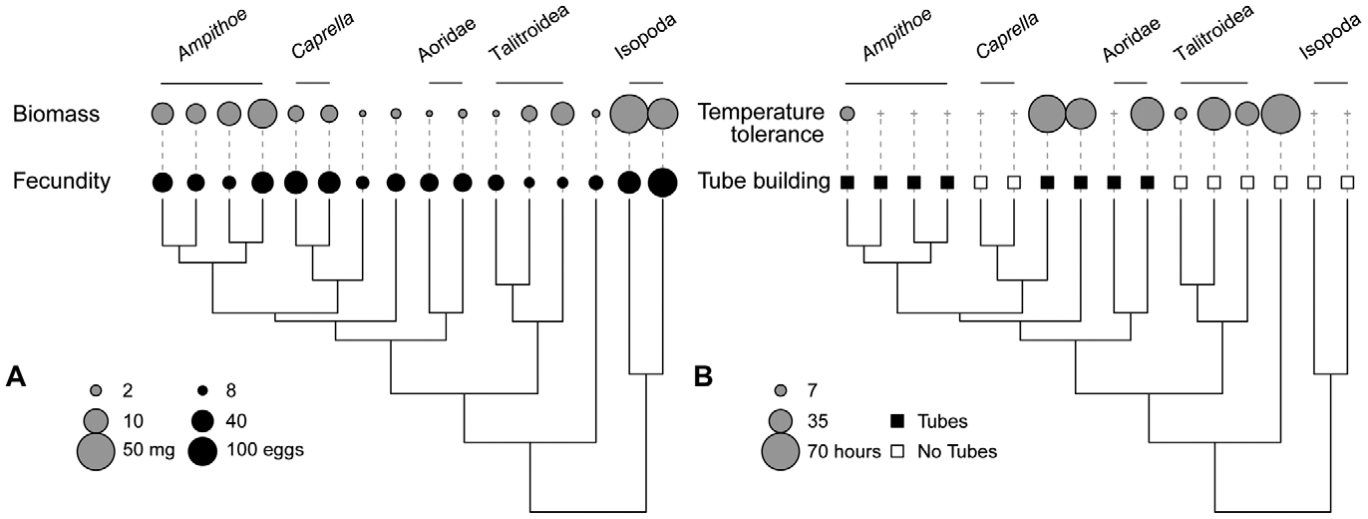
Relationship between phylogeny and A) biomass and fecundity, B) temperature tolerance and tube building. In A) larger circles represent higher biomass (dry weight) and fecundity (eggs per female) on log scales. In B) temperature tolerance is measured as the reduction in average survival time in the elevated temperature treatment (25°C) compared to controls. Larger circles indicate a larger effect of elevated temperature (i.e., lower tolerance). Species with non-significant effect of treatment (using survival analysis) have the effect set to 0 (this is indicated by a+sign). Additional figures with both means and standard errors for each trait are available as supplementary material (Figure S1).

We found clear differences in the strength of phylogenetic signal across the ecological traits we considered, with the strongest signal in body size (biomass per individual), fecundity, and tube building (average λ and K values of at least 1 and the associated p-values under 0.05, Figure 4). Closely related species were consistently similar in their mass and fecundity, although both of these traits reached high levels in distantly related clades (e.g., Ampithoid amphipods and Isopods in the far left and right of Figure 5a, see Figure 1 for species names). Variation in adult body size and fecundity between species was also clearly much greater than within species (Figure S1), even though individuals were collected throughout the year and clutch size is known to vary seasonally in amphipods [44], [45]. Tube building is perhaps the most conserved trait; it arose once with the suborder Corophiidea and was subsequently lost in the morphologically divergent Caprellidae (Figure 5b).

**Figure 6:**
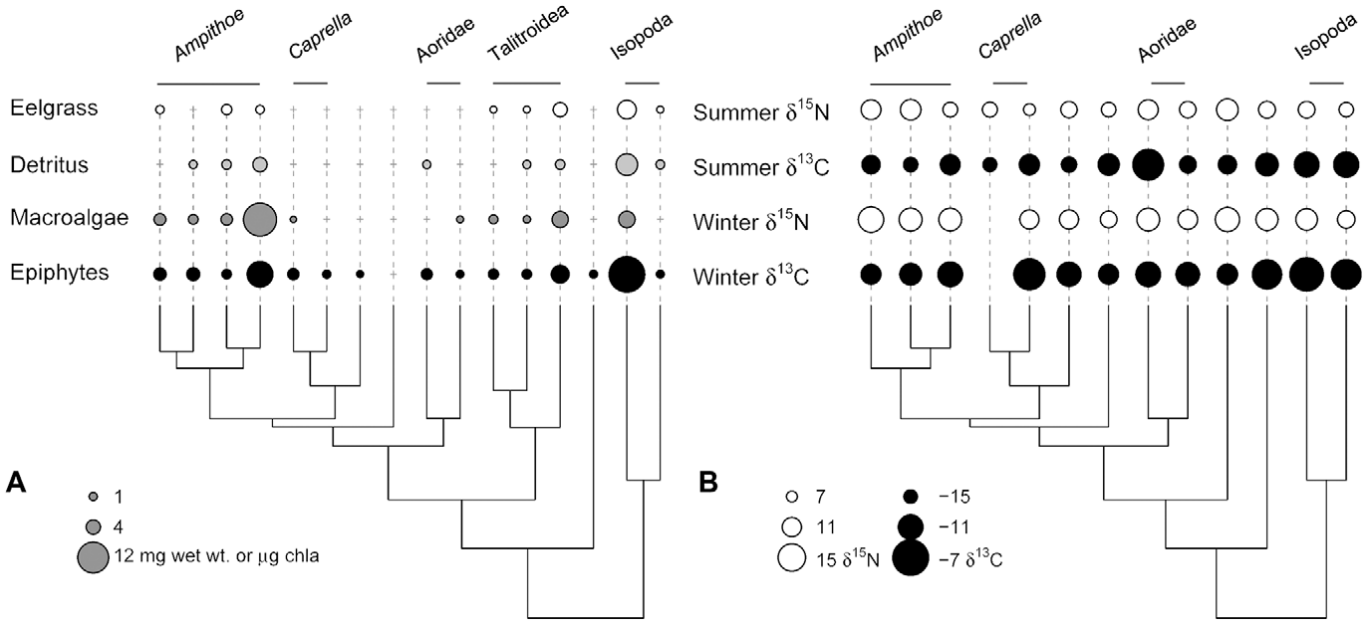
Relationship between phylogeny and A) feeding rates, B) stable isotope signatures. In A) circle size indicates feeding rates (species with non-significant feeding rates on a particular food [compared to controls] have that rate set to 0; this is indicated by a+sign). Feeding rates for eelgrass, detritus, and macroalgae (*Ulva* spp.) were measured in mg wet weight consumed per individual per day, so circle size is comparable between those foods. Feeding rates on epiphytes were measured in µg chla per individual per day, and so are not on the same scale as the other 3 foods. In B), larger circles indicate higher δ^13^C or δ^15^N. The scale is comparable across seasons within a single isotope only (i.e., N or C). Note that the 3 species found on the outer coast have been trimmed from the phylogeny and excluded from the analysis of phylogenetic signal. The species with missing winter values is present only in the summer. Additional figures with both means and standard errors for each trait are available as supplementary material (Figure S1).

In contrast, we found much less phylogenetic signal in traits related to temperature tolerance (Figure 5b), although this result is sensitive to species pool: if only amphipods are considered there is significant evidence of phylogenetic signal in temperature tolerance (Table S2, for all other traits, the relative differences in signal held even when we limited the species pool). There was also little evidence of phylogenetic signal in diet (Figure 6), especially in the feeding rates on macroalgae and epiphytes (Figure 4a). Overall, feeding niche varied substantially between close relatives as well as converging between distant relatives. Within the Ampithoids, the Talitroidea (*Allorchestes*, *Parallorchestes*, and *Protohyale*), and the Isopods some species had high feeding rates on all possible foods, and others consumed fewer foods at lower rates (Figure 6a). Because variation in feeding rates among species may be partly due to their variation in size (biomass was positively correlated with feeding rates on eelgrass, detritus, and epiphytes), we also tested for phylogenetic signal in feeding rates expressed in amount eaten per mg of grazer, rather than per individual grazer. For most foods (eelgrass, detritus, and microalgae), K and λ decreased and p-values increased in comparison to the per-individual results, suggesting that any amount of signal in those feeding rates is largely explained by body size, (see Figure 4 caption for comparative results). Along with the fact that feeding rates are correlated with biomass but show much less phylogenetic signal than biomass, this indicates that species deviate from the constraints that body size places on their potential feeding rates in ways that are unrelated to phylogeny. Finally, tests for phylogenetic signal in the discrete form of the feeding rates (eats/does not eat each food) also failed to detect any effect of shared history on feeding niche. Although the fitted value of λ was 1 for all three of eelgrass, macroalgae, and detritus, p-values were very high (Figure 4b) indicating that the ability of the phylogeny to predict feeding on these foods is not significant. All but one of the grazers consume epiphytes (Figure 6a).

**Figure 7:**
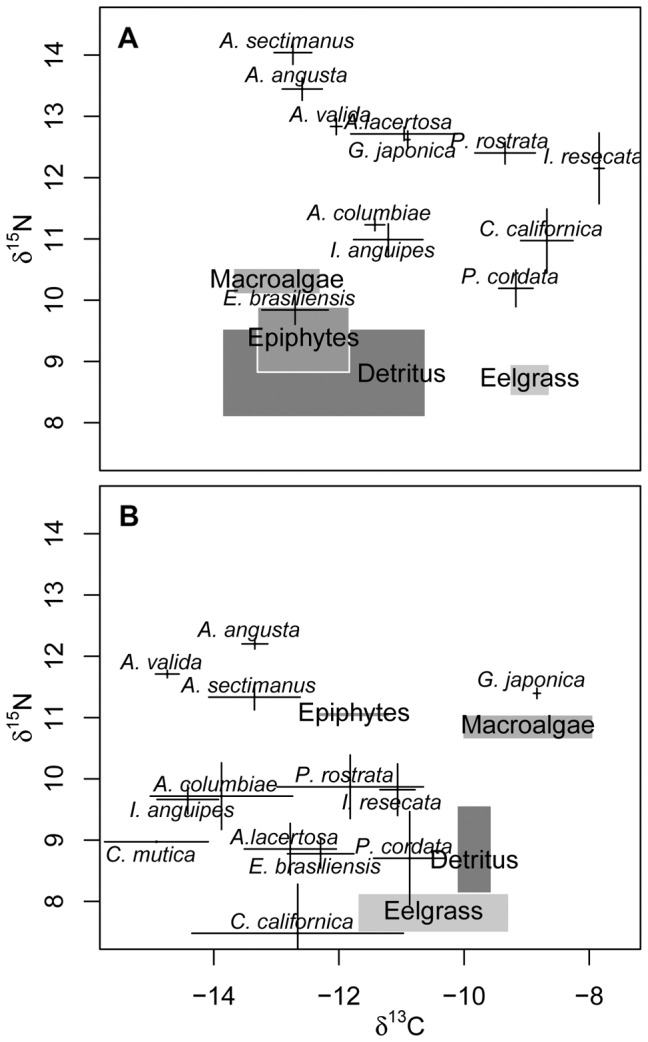
Stable isotope signatures. Grazers are represented with error bars (±1 Standard Error [SE]) and primary producers with shaded boxes (±1SE). Panels show signatures in A) winter and B) summer.

There was considerable variation among grazer species in their stable isotope signatures, potentially indicating distinct diets in the field (Figures 6b, 7). In addition, low phylogenetic signal in most of the isotopic signatures suggests that realized feeding niche is as labile as the potential feeding niche measured in laboratory feeding trials (Figure 4a). However, the relationship between stable isotope signatures in the grazers and stable isotope signatures in the primary producers is not clear and consistent, and so the interpretation of these values as reflecting realized diet from a common pool of foods is tenuous. In the winter sampling there was separation in δ^13^C values between eelgrass and algal food sources that appeared to be reflected by feeding differences in some grazers (Figure 7a); for example *I. resecata* and *P. cordata* are found in eelgrass beds and readily consume eelgrass, whereas *A. sectimanus*, *A. angusta*, and *A. valida* are found in habitats with abundant macroalgae (*Ulva* spp.) and have high feeding rates on macroalgae in the lab. *A. lacertosa* is found and was collected in both of these types of habitats, which may explain the high variance in δ^13^C for that species and its generally intermediate position between eelgrass and algal sources. In addition, the winter δ^13^C values for grazers did show some phylogenetic signal (about as much as feeding rates on eelgrass, Figure 4a). Ampithoid species had winter δ^13^C values that were more consistent (Figure 6b) than their feeding rates on any particular food or their overall potential niche (Figure 6a), as did several other closely related species. However, in summer there was poor separation among the food sources in δ^13^C, and grazers were generally more depleted in δ^13^C relative to any of the primary producers (Figure 7b). One possible explanation for this is that grazers consumed summer blooms of pelagic phytoplankton (or their detritus), which often have more negative stable isotope signatures because they experience less boundary layer resource limitation than benthic primary producers [74]. Another possibility is that the algal isotope signatures vary throughout the summer, and have summer turnover times that are much shorter than those of their consumers. This is consistent with the fact that these faster-growing producers were much more variable between seasons than the slower-growing eelgrass. If this is the case, continuous sampling over the spring and early summer might have revealed a range of δ^13^C and δ^15^N values for the algal producers that was more in alignment with the grazer signatures. Whatever the reason for seasonal variation in signatures, there was little phylogenetic signal in the summer δ^13^C signatures (or in the δ^15^N signatures from either season, Figures 4a, 6b). Although δ^15^N signatures in summer are unexpectedly low in the grazers relative to the sampled primary producers, grazer δ^15^N values are strongly correlated across seasons (r = 0.76, p = 0.004) and the δ^13^C values are somewhat correlated (r = 0.45, p = 0.13). Overall, some consistency in grazer signatures across seasons suggests that stable isotope signatures may reflect some species-specific trait variation, but the extreme variation in primary producer signals makes it difficult to link this to diet.

### Implications of Variation in Phylogenetic Signal among Traits

As described above, traits varied widely in the degree to which they show phylogenetic signal, with body mass, fecundity, and tube building showing very strong phylogenetic signal, and temperature tolerance and feeding traits showing much less (Figure 4). Thus degree of signal was not predictable based on whether the trait was more related to environmental tolerances vs. resource use, but traits related to morphology or life history were better conserved than those having to do with environmental tolerances, behavior, or resource use, as suggested by others for a range of taxa [14], [15], [75]. Because our study was not designed to sample a particular clade exhaustively, our ability to offer detailed discussion of the evolution of each of these traits is limited. Instead, our taxon sampling focused on exhaustively sampling the peracarid fauna of particular seagrass and algae ecosystems for the purposes of characterizing the phylogeny as a proxy for traits in community ecology studies. Rather than discuss the mode or rate of evolution for these traits, we thus focus our discussion on the implications of our findings about the relative strength of phylogenetic signal among traits for studies of community assembly and ecosystem function.

### Implications for Community Assembly

Understanding which types of traits have stronger phylogenetic signal is critical for interpreting patterns of phylogenetic community structure. Traits determining the β-niche (e.g., environmental tolerances, macro-habitat requirements) often show different evolutionary patterns than those determining the α-niche (e.g., resource partitioning, micro-habitat use), because coexisting species must evolve similarities in the former and differences in the latter [11], [12], [14], [76], [77], [78]. In our study, however, we found that phylogenetic signal varies not only between the broad categories of β-niche and α-niche traits, but also within those categories.

In our system, habitats vary at a coarse scale in both water temperature (with shallower habitats reaching higher water temperatures at low tide), and habitat availability (seagrass, macroalgae, or both). Potential determinants of the β-niche thus include both temperature tolerance, which is labile if isopods are included in the species pool (Table S2), and preferences for different types of habitat, which may be influenced by more conserved traits: body size [79], [80] and the ability to construct tubes. The choice of host plants as habitat also appears to be genus-specific in at least one family of tube builders, indicating additional phylogenetic signal in habitat use [81]. Because trait conservatism varies between environmental-filtering and habitat-filtering traits, closely related species may or may not share the trait values necessary to survive in a given set of conditions.

Similarly, traits that determine a species’ α-niche also vary in their evolutionary lability.

If tube-building ability and size determine micro-habitat as well as macro-habitat (for example, species of amphipods in the genus *Gammarus* segregate microhabitat according to body size [80]), or if there is α-niche differentiation in fecundity, where species produce different numbers of offspring varying in their growth rate vs. competitive ability, then phylogenetic relatedness may indicate niche overlap. However, phylogeny is a poor indicator of overlap in either fundamental or realized feeding niche (measured via feeding trials and stable isotope signatures, respectively). Consistent with this, we found no link between the effects of feeding trait diversity and phylogenetic diversity on the outcome of resource competition in mesocosms [82]. The lack of correspondence between phylogeny and feeding traits could be due to the evolution of mouthparts, physiology, and/or behavior, and previous work has shown that amphipod mouthparts [83] and physiological tolerances [84] can vary independently of phylogeny (although this can be due to a lack of evolutionary change [83] or to convergent evolutionary change). Overall, the implication for community assembly is that closely related species of amphipods and isopods are not more likely to compete for food, only for micro-habitat. This is in contrast to the evolution of physiological tolerances in many terrestrial insects, where host use for both food and habitat is more strongly conserved [81], [85], and again complicates the interpretation of phylogenetic community structure in the field.

### Implications for Ecosystem Functioning

If the traits that determine species’ effects on ecosystem processes are conserved, then the phylogenetic relatedness of a community can be used to predict ecosystem function, either via complementarity or dominance [4], [5]. As discussed above, phylogenetic distance is a poor proxy for complementarity in feeding niche, and therefore unlikely to predict whether a community of mesograzers is able to perform the key function of removing multiple types of algal competitors to promote seagrass growth (see for example [86]). The very strong phylogenetic signal in body mass, however, could link phylogeny to ecosystem function because large species frequently have large impacts on function [4], [87], [88]. For example, we have found that the presence or absence of the largest amphipod in our system (*Ampithoe lacertosa*) can have a much larger impact on algal abundance than the resource complementarity of the grazer community [82]. Here, though, it seems that phylogenetic signal is not the only characteristic of trait evolution that matters for inference. The strong phylogenetic signal in body mass indicates that closely related species are more similar in size, perhaps even more-so than we would expect from evolution via Brownian motion. This does not, however, rule out convergent evolution. Large body size has evolved in both amphipods and isopods, and even within our pool of 14 amphipod species there are multiple families with large-bodied species. Thus complete sampling within a particular clade (low phylogenetic diversity) and even sampling across many clades (high phylogenetic diversity) might be equally likely to include a large bodied (high-impact) species, leading to no clear relationship between phylogenetic diversity and ecosystem functioning.

### Conclusions

In this study, we set out to resolve phylogenetic relationships between our species, measure a range of potentially important traits, and compare the degree to which each of those traits are evolutionarily conserved. Despite areas of very poor phylogenetic resolution in the broader amphipod phylogeny, we were able to resolve almost all of the topology connecting the species in our system. For studies of amphipod communities elsewhere, the ease of obtaining phylogenetic relationships will therefore depend on the sampling of species. If species pools have representatives from multiple families in the Corophiidean suborder and only a few families in the Gammaridea, as is typical in many seagrass systems, phylogenies constructed from the genes used here (COI, 16S, and 18S), may provide adequate resolution for use in studies of community processes. Otherwise, much additional effort will need to be devoted to developing new molecular markers that better capture the evolutionary relationships between families.

As expected, we found substantial variation in phylogenetic signal among the traits we measured. Strong signal in body size, fecundity, and tube building suggests that phylogenies may be good proxies for some types of habitat use and demographic niche. Conversely, weaker signal in feeding traits and temperature tolerance indicate that phylogenetic patterns should not be interpreted as evidence of environmental filtering along water temperature gradients or of the potential complementarity of feeding niches. This means that trait lability in our species varies among, as well as between, potential α and β-niche traits, complicating the interpretation of phylogenetic community structure and reducing the predictive potential of phylogenetic proxies in the absence of detailed trait data.

## Supporting Information

Figure S1 **Trait data.** Panels A to K show mean trait values for each species (±1 Standard Error). Non-significant outcomes of laboratory experiments (e.g., temperature trials in panel C, feeding trials in panels D-G) are shown as 0. Panel C shows the reduction in survival time (in hours) in elevated water temperature (25°C) relative to controls.
(PDF)

Table S1 **Additional 18S sequences.**
(PDF)

Table S2 **Results of tests for phylogenetic signal with a subset of species.** We report Blomberg’s K for continuous traits and Pagel’s λ for discrete traits, plus the p-value for the corresponding significance test (H0: signal no greater than 0). All values are averaged over 200 trees sampled from the posterior distribution. The power to detect significant signal decreases with the number of species in the pool, but the relative amount of signal between traits is consistent (except for temperature tolerance when isopods are excluded). P-values <0.05 are italicized, along with their corresponding K or λ values.
(PDF)
