# Supplementary figures and images for: Phylogeny as a Proxy for Ecology in Seagrass Amphipods: Which Traits Are Most Conserved?
Source: PLoS One. 2013 Mar 7;8(3):e57550. doi: 10.1371/journal.pone.0057550 (PMC3591422; doi:10.1371/journal.pone.0057550)

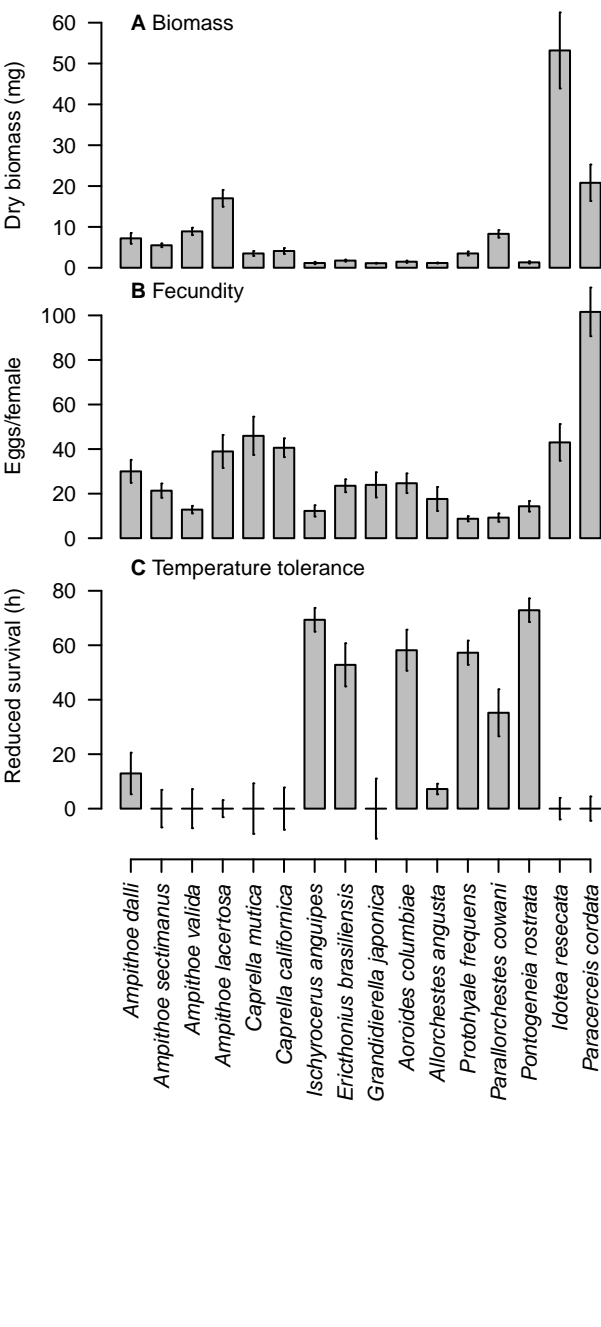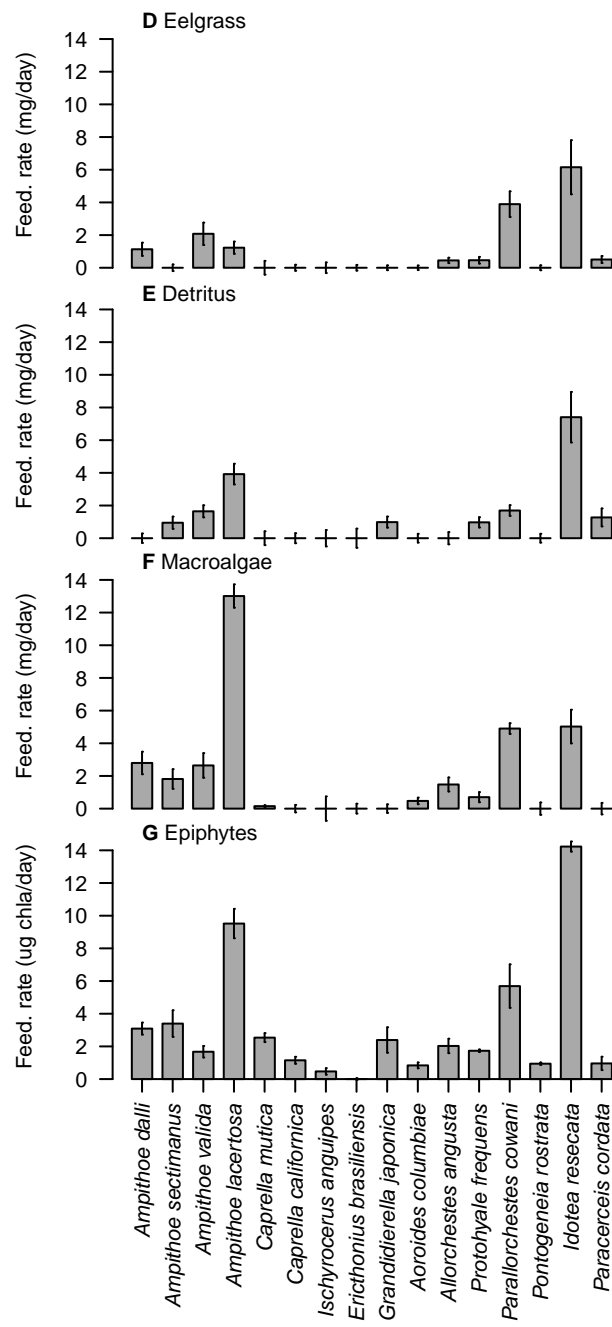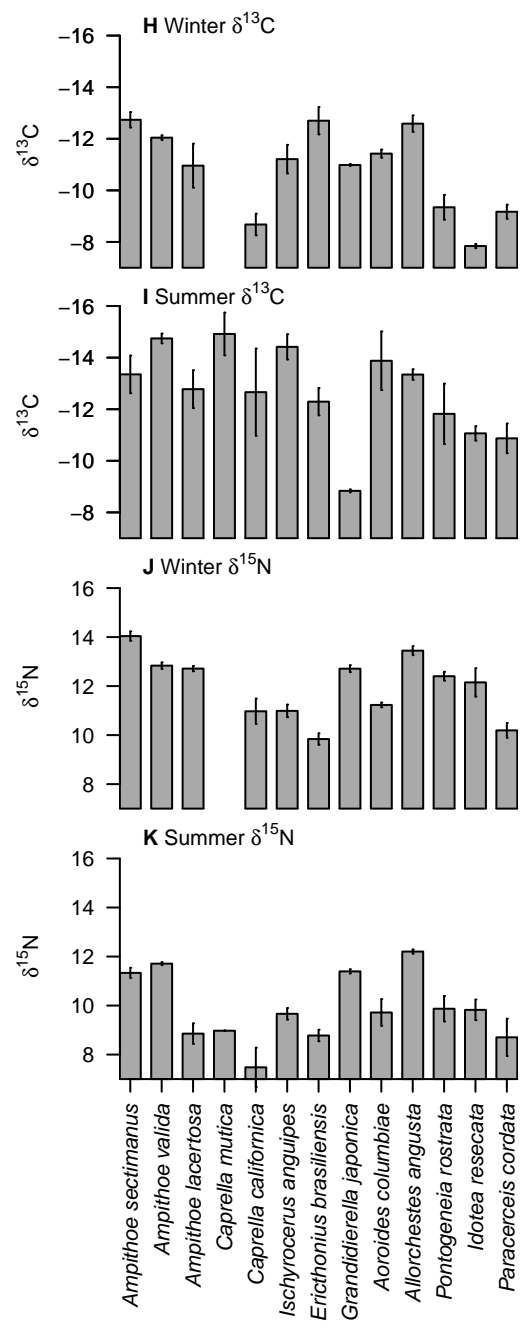

Supplement: Figure S1 — Trait data. Panels A to K show mean trait values for each species (±1 Standard Error). Non-significant outcomes of laboratory experiments (e.g., temperature trials in panel C, feeding trials in panels D-G) are shown as 0. Panel C shows the reduction in survival time (in hours) in elevated water temperature (25°C) relative to controls. (PDF) [file pone.0057550.s001.pdf]
