# Supplementary material for: Phylogeny as a Proxy for Ecology in Seagrass Amphipods: Which Traits Are Most Conserved?
Source: PLoS One. 2013 Mar 7;8(3):e57550. doi: 10.1371/journal.pone.0057550 (PMC3591422; doi:10.1371/journal.pone.0057550)
Supplement: Table S1 — Additional 18S sequences. (PDF) [file pone.0057550.s002.pdf]

Family	Species	Accession #s	Collection Locality
Suborder: Corophiidea			
Ampithoidae	*Ampithoe rubricata*	DQ378023[1]	Norway: Porsanger
Ampithoidae	*Ampithoe longimana*	AY926757, AY926819[2]	USA: Chesapeake Bay
Ampithoidae	*Ampithoe ramondi*	DQ378024[1]	Curacao
Ampithoidae	*Ampithoe lacertosa*	AB295402[3]	Japan: Shizuoka
Aoridae	*Grandidierella japonica*	AB295403[3]	Japan:Chiba
Aoridae	*Aora gracilis*	DQ378019[1]	Baltic Sea
Unciolidae*	*Neohela monstrosa*	DQ378018[1]	Baltic Sea
Caprellidae	*Phtisica marina*	DQ378040[1]	?
Caprellidae	*Caprella linearis*	DQ378039[1]	Baltic Sea
Caprellidae	*Pseudoprotella phasma*	DQ378041[1]	Baltic Sea
Caprellidae	*Caprella danilevskii*	AB295398[3]	Japan: Kumamoto
Caprellidae	*Protella gracilis*	AB295396[3]	Japan: Kumamoto
Caprellidae	*Monoliropus tener*	AB295395[3]	Japan: Shizuoka
Caprellidae	*Caprella geometrica*	AY781423[4]	USA: Massachusetts
Corophiidae	*Corophium volutator*	DQ378027[1]	Scotland: Cumbrae
Corophiidae	*Bubocorophium sp. AI-2007-6*	AB295405[3]	Japan: Shizuoka
Corophiidae	*Corophium sp. AI-2007-5*	AB295404[3]	Japan: Shizuoka
Dulichiidae	*Dulichia porrecta*	DQ378020[1]	Norway: Porsanger
Isaeidae	*Gammaropsis utinomii*	AB295406[3]	Japan: Kumamoto
Isaeidae	*Gammaropsis melanops*	DQ378028[1]	Norway: Porsanger
Ischyroceridae	*Ericthonius pugnax*	AB295407[3]	Japan: Shizuoka
Ischyroceridae	*Ericthonius brasiliensis*	DQ378016[1]	Baltic Sea
Ischyroceridae	*Jassa falcata*	DQ378017[1]	Baltic Sea
Podoceridae	*Podocerus septemcarinatus*	DQ378021[1]	?
Suborder: Gammaridea			
Ampeliscidae	*Ampelisca eschrichti*	AY826963[1]	Norway: Porsanger
Ampeliscidae	*Byblis gaimardi*	AY826964[1]	Norway: Porsanger
Anisogammaridae	*Eogammarus oclairi*	AY926768, AY926830[2]	Canada: Vancouver Island
Anisogammaridae	*Jesogammarus debilis*	EF582934[5]	China: Beijing
Atylidae	*Atylus swammerdami*	DQ378031[1]	Baltic Sea
Calliopiidae	*Apherusa bispinosa*	DQ378009[1]	Germany: North Sea
Calliopiidae	*Calliopius laeviusculus*	DQ378008[1]	Germany: North Sea
Colomastigidae*	*Colomastix fissilingua*	DQ378032[1]	?
Crangonyctidae	*Bactrurus brachycaudus*	AF202979[6]	USA: Missouri
Crangonyctidae	*Stygobromus mackini*	DQ377995[1]	?
Crangonyctidae	*Crangonyx pseudogracilis*	EF582897[5]	Canada: Ontario
Epimeriidae*	*Epimeriella walkeri*	DQ378005[1]	?
Epimeriidae*	*Epimeria georgiana*	AF356546[1]	Antarctica
Eusiridae	*Eusirus perdentatus*	DQ378012[1]	Antarctica
Gammarellidae*	*Gammarellus homari*	DQ378033[1]	Baltic Sea
Gammaridae	*Chaetogammarus marinus*	AY926760, AY926822[2]	Norway: Bergin
Gammaridae	*Dikerogammarus villosus*	EF582898[5]	Germany: Brandenburg
Gammaridae	*Gammarus annulatus*	AY926779, AY926841[2]	USA: Massachusetts
Gammaridae	*Gammarus oceanicus*	AY781422[5]	USA: Massachusetts
Gammaridae	*Gammarus locusta*	AF419222[7]	Baltic Sea
Gammaridae	*Sinogammarus chuanhui*	EF582937[5]	China: Guizhou
Haustoriidae	*Haustorius arenarius*	AY826950[1]	Scotland: Cumbrae
Hyalidae	*Parhyale hawaiensis*	AY826957[1]	Curacao

Family	Species	Accession #s	Collection Locality
Hyalidae	*Hyale nilssoni*	AY826958[1]	Scotland: Cumbrae
Iphimediidae	*Echiniphimedia hodgsoni*	DQ378004[1]	Antarctica
Iphimediidae	*Iphimediella georgei*	DQ378002[1]	Antarctica
Leucothoidae	*Leucothoe spinicarpa*	DQ378025[1]	?
Liljeborgiidae	*Lilleborgia fissicornis*	AY826959[1]	Norway: Vargsund
Eurytheneidae	*Eurythenes gryllus*	AY826967[1]	Antarctica
Uristidae	*Tryphosella murrayi*	AY826965[1]	Antarctica
Lysianassidae	*Lepidepecreum umbo*	AY826968[1]	Norway: Porsanger
Uristidae	*Menigrates obtusifrons*	AY826966[1]	Norway: Porsanger
Maeridae	*Maera inaequipes*	AF419229[7]	Spain: Roses
Maeridae	*Paraceradocus gibber*	AF419232[7]	Antarctica
Megaluropidae	*Megaluropus longimerus*	DQ378035[1]	Curacao
Melitidae	*Melita nitida*	AY926795, AY926856[2]	USA: Chesapeake Bay
Melitidae	*Megomaera subtener*	AY926794, AY926855[2]	USA: Washington
Melphidippidae	*Melphidippa borealis*	DQ377997[1]	?
Pleustidae	*Pleustes panoplus*	DQ378034[1]	Norway
Pontogammaridae*	*Pontogammarus crossus*	AY926811, AY926872[2]	Black Sea
Pontoporeiidae	*Bathyporeia pilosa*	AY826951[1]	Scotland: Cumbrae
Pontoporeiidae	*Monoporeia affinis*	AY926800, AY926861[2]	?
Stegocephalidae*	*Stegocephalus inflatus*	AY826970[1]	Norway: Porsanger
Stegocephalidae*	*Andaniexis lupus*	AY826969[1]	Norway: Vargsund
Stenothoidae	*Stenothoe brevicornis*	AY826962[1]	Norway: Porsanger
Stenothoidae	*Antatelson walkeri*	AY826961[1]	Antarctica
Stilipedidae	*Astyra abyssi*	DQ378000[1]	Norway: Porsanger
Talitridae	*Orchestia gammarellus*	AY826954[1]	Scotland: Cumbrae
Talitridae	*Talitrus saltator*	AY826955[1]	Scotland: Cumbrae
Urothoidae	*Urothoe brevicornis*	AY826973[1]	Scotland: Cumbrae
Additional Bodega Bay Gammaridean sequences without trait data			
Calliopiidae	*Oligochinus lighti*	JX545408, JX545372**	Bodega Bay, outer coast
Melitidae	*Megamoera subtener*	JX545406, JX545370**	Bodega Bay, eelgrass bed

* Family not represented in Northern California region [8].

** Sequenced for this study.

1. Englisch U (2001) Analyse der Phylogenie der Amphipoda (Crustacea, Malacostraca) mit Hilfe von Sequenzen des Gens der RNA der kleinen ribosomalen untereinheit. PhD Thesis. Ruhr–Universität Bochum.
2. Macdonald KS, Yampolsky L, Duffy JE (2005) Molecular and morphological evolution of the amphipod radiation of Lake Baikal. Mol Phylogenet Evol 35: 323-343.
3. Ito A, Wada H, Aoki MN (2008) Phylogenetic analysis of caprellid and corophioid amphipods (Crustacea) based on the 18S rRNA gene, with special emphasis on the phylogenetic position of Phtisicidae. Biol Bull 214: 174-181.
4. Spears T, DeBry RW, Abele LG, Chodyla K, Boyko CB (2005) Peracarid monophyly and interordinal phylogeny inferred from nuclear small-subunit ribosomal DNA sequences (Crustacea: Malacostraca: Peracarida). Proc Biol Soc Wash 118: 117-157.
5. Hou ZG, Fu JH, Li SQ (2007) A molecular phylogeny of the genus Gammarus (Crustacea : Amphipoda) based on mitochondrial and nuclear gene sequences. Mol Phylogenet Evol 45: 596-611.
6. Englisch U, Koenemann S (2001) Preliminary phylogenetic analysis of selected subterranean amphipod crustaceans, using small subunit rDNA gene sequences. Org Div Evol 1: 139-145.
7. Englisch U, Coleman CO, Waegele JW (2003) First observations on the phylogeny of the families Gammaridae, Crangonyctidae, Melitidae, Niphargidae, Megaluropidae and Oedicerotidae (Amphipoda, Crustacea), using small subunit rDNA gene sequences. J Nat Hist 37: 2461-2486.
8. Chapman JW (2007) Gammaridea. In: Carlton JT, editor. The Light and Smith Manual: Intertidal invertebrates from central California to Oregon. Berkeley: UC Press. pp. 545-618.
