# Supplementary material for: Phylogeny as a Proxy for Ecology in Seagrass Amphipods: Which Traits Are Most Conserved?
Source: PLoS One. 2013 Mar 7;8(3):e57550. doi: 10.1371/journal.pone.0057550 (PMC3591422; doi:10.1371/journal.pone.0057550)
Supplement: Table S2 — Results of tests for phylogenetic signal with a subset of species. We report Blomberg’s K for continuous traits and Pagel’s λ for discrete traits, plus the p-value for the corresponding significance test (H0: signal no greater than 0). All values are averaged over 200 trees sampled from the posterior distribution. The power to detect significant signal decreases with the number of species in the pool, but the relative amount of signal between traits is consistent (except for temperature tolerance when isopods are excluded). P-values <0.05 are italicized, along with their corresponding K or λ values. (PDF) [file pone.0057550.s003.pdf]

	All species (16)		Harbor species only (13)		Amphipods only (14)	
**Continuous trait**	**K**	**p**	**K**	**p**	**K**	**p**
Biomass	*1.72*	*0.01*	*1.55*	*0.02*	0.70	0.21
Fecundity	*1.10*	*0.04*	0.98	0.10	0.68	0.20
Eelgrass	0.90	0.07	0.99	0.15	0.71	0.22
Detritus	0.84	0.12	0.88	0.13	0.49	0.59
Epiphytes	0.61	0.26	0.60	0.33	0.46	0.64
Temperature	0.58	0.19	0.59	0.24	*0.91*	*0.04*
Macroalgae	0.37	0.68	0.37	0.69	0.48	0.59
**Discrete trait**	**λ**	**p**	**λ**	**p**	**λ**	**p**
Tube building	*1.00*	*0.03*	1.00	0.14	*1.00*	*0.05*
Eelgrass	0.98	0.25	0.95	0.28	0.97	0.26
Macroalgae	0.97	0.36	0.66	0.77	0.97	0.34
Detritus	0.82	0.70	1.00	0.19	0.84	0.70
